# The Effect of Training on Postural Control in Dyslexic Children

**DOI:** 10.1371/journal.pone.0130196

**Published:** 2015-07-10

**Authors:** Nathalie Goulème, Christophe-Loïc Gérard, Maria Pia Bucci

**Affiliations:** 1 UMR 1141 Inserm—Paris Diderot University, Robert Debré Hospital, Paris, France; 2 Child and Adolescent Psychiatry Department, Robert Debré Hospital, Paris, France; University of Medicine & Dentistry of NJ—New Jersey Medical School, UNITED STATES

## Abstract

The aim of this study was to explore whether a short postural training period could affect postural stability in dyslexic children. Postural performances were evaluated using Multitest Equilibre from Framiral. Posture was recorded in three different viewing conditions (eyes open fixating a target, eyes closed and eyes open with perturbed vision) and in two different postural conditions (on stable and unstable support). Two groups of dyslexic children participated in the study, i.e. G1: 16 dyslexic participants (mean age 9.9 ± 0.3 years) who performed short postural training and G2: 16 dyslexic participants of similar ages (mean age 9.1 ± 0.3 years) who did not perform any short postural training. Findings showed that short postural training improved postural stability on unstable support surfaces with perturbed vision: indeed the surface, the mean velocity of CoP and the spectral power indices in both directions decreased significantly, and the cancelling time in the antero-posterior direction improved significantly. Such improvement could be due to brain plasticity, which allows better performance in sensory process and cerebellar integration.

## Introduction

According to the American Psychiatric Association [1], dyslexia is a neurobiological disorder characterized by a difficulty in reading acquisition despite adequate intelligence and conventional education, motivation or social level.

According to an exhaustive literature, dyslexic children have poor postural control with respect to control age-matched children. Postural control abilities in humans depend on the capacities to detect the environment and visual, vestibular and somatosensory inputs are used to obtain a good quality of postural control. A cerebellar integration allows a weighing of sensory information to achieve postural stability [2].

Several studies describe poor motor coordination, which could be due to cerebellar deficit in dyslexic children [3–5]. Many symptoms observed in dyslexia have also conducted scientists to suspect a cerebellar origin [6]. This deficit could be associated to a delay of maturity in the cerebellar development, as described by Stoodley et al. [7]. Also, Konczak et al. [8] found that dyslexic children display poor motor performances similar to those reported in children with cerebellar lesions. Another study from O’hare et al. [9] describes that such children have a deficit in motor coordination, suggesting a cerebellar syndrome. Several studies [10–14] highlight the fact that dyslexic children show poorer postural performances compared to non-dyslexic children during a dual task paradigm, suggesting poor automaticity capabilities in dyslexia. This hypothesis is in line with the work conducted by Viana et al. [15], who compared postural control in dyslexic and non-dyslexic age-matched children using a moving room and making subjects manipulate somatosensory information. They showed that dyslexic children were more unstable than non-dyslexic children and that in sensory-perturbed conditions dyslexic children did not compensate as well as non-dyslexic children. Thus suggesting that dyslexic children could have a deficit in multisensory integration of multiple inputs. All of these findings are in line with our recent study exploring postural capabilities in dyslexic children by using wavelet transformation, suggesting that poor postural performance observed in dyslexic children could be due to a poorer use of sensory inputs and a lack of cerebellar integration [16]. This hypothesis is in line with other neurophysiological studies. Indeed, Rae et al. [17], using magnetic resonance spectroscopy in adult dyslexic participants, reported that there were biomechanical differences between dyslexic and control participants in the left temporo-parietal lobe and right cerebellum. They also noted lateral differences in these regions, suggesting an altered cerebral structure and an abnormal cortical development in dyslexic participants. Moreover, Eckert et al. [18] using MRI scans, described a significantly smaller right anterior lobe of the cerebellum and brain volume in dyslexic children compared to control age-matched children, suggesting that the cerebellum could be one of the most significant locations involved for structural differences between dyslexic and non-dyslexic children.

The cerebellum could be under adaptive mechanisms; indeed, Sehm et al. [19] described an improvement in postural performance after postural training on movable support in healthy participants as well as in patients with Parkinson’s disease. Interestingly, such improvement was correlated with a change of grey matter in the right cerebellum, suggesting brain plasticity capabilities. Moreover, Burciu et al. [20] applied structural magnetic resonance imaging in 20 patients with cerebellar degeneration after two weeks of postural training. This training consisted in maintaining their body in a specific area during 10 seconds, which could be controlled on a PC screen. These authors reported both an improvement in postural performances and an increase of gray matter volume in the dorsal pre-motor cortex. Another study led by Abd El-Kafy et al. [21] described the effect of eight weeks of training (2 hours, three times per week) in a group of thirty children from 8 to 10 years with cerebral palsy. The training was traditional physiotherapy only for half of the children, and for the other half of the children it consisted in traditional physiotherapy associated with a dynamic postural training program. Both groups of children showed an improvement of performance in postural control and in gait pattern; however, the group with the training associated with a dynamic postural training program had a significantly better performance, suggesting the relevant role of dynamic postural training. These authors made the hypothesis that cortical plasticity could be responsible for developing neural connectivity, and thus for improving postural control. At our knowledge, studies leading with short postural training in children are scarce. Katz-Leurer et al. [22] evaluated in children with cerebral palsy or post traumatic brain injury the effect of a short training period (one minute each day only) for five days per week for six weeks. The exercise program consisted to sit-to-stand and step-up as many times as possible without any external assistance or use of the hands. The authors showed that training improved postural performance most likely due to cerebral plasticity. More recently, Angulo-Barroso et al. [23] analyzed the development of walking onset in younger children at risk for neuromotor delay. The authors explored in 15 children from 9.1 to 9.7 years old the effect of a treadmill training 8 minutes per day, for five days a week until walking onset and they found that children with training showed a better performance on treadmill and on quality of their step, suggesting that postural training could improve the motor development by cortical activities even in children with developmental delay.

The present study explored whether a short postural training period could have an effect on the postural capabilities of dyslexic children. Our hypothesis was that, due to cerebellum plasticity, training could improve re-weighing sensory inputs in order to increase somatosensory integration and lead to better postural control. The use of wavelet transformation could allow us to gain insight into the performance of different sensory inputs in dyslexic children. Thus, based on previous findings, we expected to find an improvement of both spatial and temporal postural parameters after training, suggesting a better use of sensory inputs, an improvement that could be linked to cerebellum plasticity in dyslexic children.

## Methods

### Participants

Dyslexic participants were randomly divided into two groups in our current study: one group (G1) of 16 dyslexic participants (mean age 9.9 ± 0.3 years) who had a short postural training and another group (G2) of 16 dyslexic participants (mean age 9.1 ± 0.3 years), without any postural training. All participants had neither drug treatment nor any orthopedic lesion.

Dyslexic participants were recruited from a pediatric hospital to which they had been referred for a full evaluation of their dyslexia, including neurological/psychological and phonological capabilities. For each participant, the time required to read a text passage was measured, assessed general text comprehension, and evaluated the ability to read words and pseudo-words using the L2MA battery [24]. This is the standard test developed by the Centre de Psychologie appliquée de Paris, often used in France and already employed in our previous studies for selecting dyslexic population [25]. Inclusion criteria were: scores to this test beyond 1.5 standard deviations, and a normal mean intelligence quotient (IQ, evaluated with WISC-IV; between 85 and 115). At least in France, a child is considered to be dyslexic when her/his reading capabilities are delayed at least beyond 1.5 standard deviations with respect to reading-age matched children. Mean IQ and mean reading age were 103 ± 1.1 and 7.4 ± 0.2 year respectively for Group G1 and 98 ± 1.4 and 7.3 ± 0.5 year for Group G2.

The investigation adhered to the principles of the Declaration of Helsinki and was approved by our Institutional Human Experimentation Committee (Comité de Protection des Personnes CPP Ile de France V, Hôpital Saint-Antoine). Written informed consent was obtained from the participants’ parents after the nature of the procedure had been explained.

### Postural recording procedure

Multitest Equilibre from Framiral has been used to evaluate postural performance. This Multitest Equilibre involved a support static and dynamic by Micromedical Technologies (www.framiral.fr). The CoP displacement was sampled at 40 Hz and 100 Hz in the static and dynamic conditions respectively, and digitized with 16-bit precision. During unstable conditions, participants were on a dynamic platform consisting of a force plate mounted on a translator that can move in the antero-posterior (y) and the medio-lateral directions (x). Moreover, a computer-controlled mechanism allowed the platform to make sinusoidal displacements of 62 mm in amplitude with adjustable velocities and frequencies.

The ramp mode allows forward and backward translations of the force plate, with constant linear velocities of 0.03 m/s and 0.07 m/s. For the sinusoidal mode, the frequency was 0.25 Hz [26–27].

The participants were in a dark room on the Framiral platform, positioned on the platform footprints, arms along the body. The dark room was used in order to avoid that visual information from the environment influence the capability of participants to control their posture. Recording was performed under three visual conditions (eyes open fixating a target: EO, eyes closed: EC and eyes open in perturbed vision with optocinetic stimulation: OPTO) on stable (S) and unstable support surfaces (U). During the eyes open condition the participants have to fixate a small red light at a distance of 250 cm; however, the eye movements were not recorded. The optocinetic stimulation was performed by an optocinetic ball that was projected on a wall at a distance of 250 cm from the participants’ eyes and turned with 158 per second angular speed [28]. The optocinetic stimulation allows to evaluate postural stability when vision is perturbed. The duration of each postural recording was 30 seconds with 15 seconds of rest between each condition to reduce possible fatigue effects. Concerning spatial analysis parameters, the surface area and mean velocity of the CoP were measured. Concerning temporal analysis parameters, the spectral power indices and the cancelling time for both medio-lateral (x) and antero-posterior (y) directions were evaluated. This temporal analysis was done for each frequency: low (L), 0.05–0.5 Hz, medium (M), 0.5–1.5 Hz, and high (H) >1.5Hz. Postural recording was done before and after short postural training period for G1 and twice for G2, which was not trained.

### Training stimulation

After the first postural recording, dyslexic participants from G1 had a five-minute rest before starting the training stimulation, which consisted in avoiding people who walked towards the child (see S1 Movie). The protocol was explained to the participants and a test of 30 seconds was conducted to ensure that the participants had understood the instructions. Then, the training duration was of 3 minutes. Participant was on the platform situated 250 cm away from a screen (340 cm x 170 cm). Passers were in colors walking in a street towards to the participant with a mean velocity ranging from 0.5 mm/s to 1.5 mm/s. The participant had to move his/her body efficiently to avoid the passers-by. After the training, the participant had a rest of a five minutes and another postural recording was done. Concerning the dyslexic particpants from G2, they were not trained but postural measure was done twice, before and after a five-minute rest.

### Classical data in the spatial domain

Both the surface area (cm^2^) and the mean velocity (mm/s) of the center of pressure (CoP) were analyzed in order to quantify postural performance. The surface area of the CoP is an efficient measure of CoP spatial variability, corresponding to an ellipse with 90% of CoP excursions [29]. The mean velocity of the CoP represents a good index of the amount of neuromuscular activity required to regulate postural control [30–31]. These two postural parameters allow efficient measurement of CoP spatial variability.

### Frequency analysis

A wavelet analysis was applied to study the frequency of the CoP displacements. This analysis and associated parameters were obtained with software from Framiral (www.framiral.fr [27; 32]).

The spectral power index was calculated as the decimal logarithm for the frequency bands 0.05–0.5 Hz, 0.5–1.5 Hz, higher than 1.5 Hz on the medio-lateral and antero-posterior directions (PIx and PIy, respectively). The spectral power index in the higher band is minimal in healthy participants during quiet standing, but it can be observed with aging, in postural pathology or in dynamic postural conditions [33]. The hypothetical physiological significance of the different bands is as follows: 0–0.5 Hz visual-vestibular [33–35], 0.5–1.5 Hz cerebellar [35] and 1.5 Hz reflexive loops [27; 36].

Moreover, the cancelling time (CT) of each frequency band was also calculated for the medio-lateral (CTx) and the antero-posterior (CTy) directions; it is the total time during which the spectral power index of the body sway for the frequency range is cancelled by the posture control mechanisms; the longer the cancelling time of a frequency band, the better the posture control [27; 32]. Cancelling time is the time required to use sensory inputs. Thus, the higher cancelling time is, the more participants use their sensory information (visual, vestibular and somesthesic inputs, respectively). A small cancelling time reveals a low quest time of the sensory inputs and thus a poor use of these to maintain postural control.

The cancelling time at a certain frequency is reduced to zero over a period of time indicates a successful action of the postural control system since the overall entropy of the sway has been reduced. While most healthy participants exhibit these zero power instances in their postural sway spectrum, pathological participants cannot. How the cancelled frequencies are ‘chosen’ by the postural control system is not known yet, but it is assumed that the choice criterion is the minimization of muscular effort required to control the sway [37]. These parameters were analyzed for the two postural measures for both groups of participants (G1 and G2).

### Statistical analysis

Statistical analysis was performed with the Statistica sotware. An analysis of variance (ANOVA) was performed on the first postural recording done for the two groups of dyslexic participants (G1 and G2) in order to be sure that their postural parameters were similar. The individual Student’s t-test was used to compare the two postural measures done. The effect of a factor was considered significant when the p-value was below 0.05.

## Results

ANOVA failed to show any statistical difference on postural parameters (surface and mean velocity of the CoP) between the two groups of dyslexic participants G1 and G2 (F_(1,30)_ = 0.33, p = 0.56 and F_(1,30)_ = 0.19, p = 0.66, respectively).

### Postural data in the spatial domain

#### Surface of the CoP

**Fig 1:**
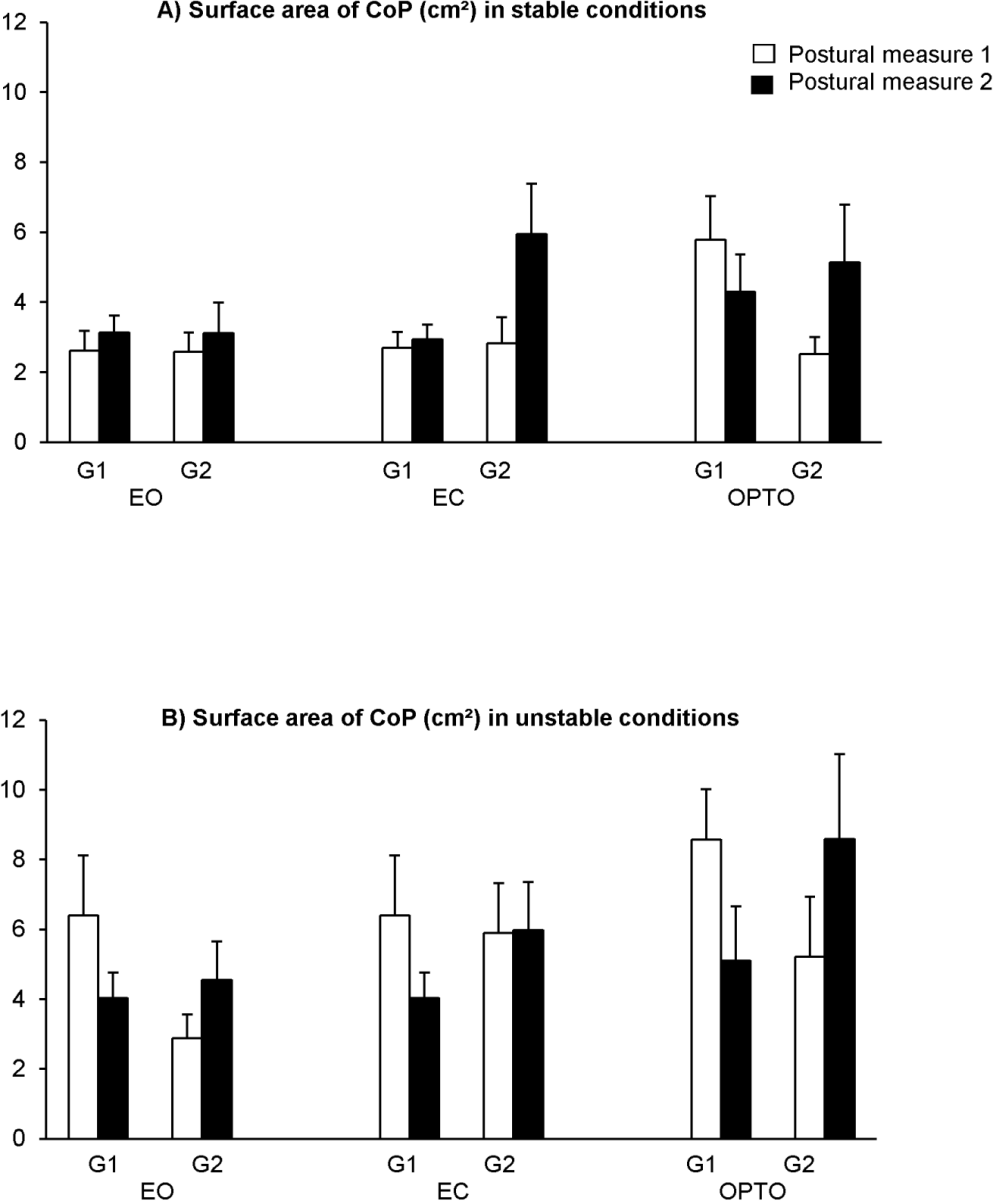
Means and standard deviations of surface area of CoP (cm^2^) in all conditions tested (EO, EC and OPTO) in stable condition (A) and in unstable condition (B) during the two postural measures (1 and 2) for both groups of children with (G1) and without training (G2).

Fig 1 shows the surface area of the CoP (cm^2^) in all conditions tested (EO, EC and OPTO) in stable condition (Fig 1A) and in unstable condition (Fig 1B) during the two postural measures (1 and 2) for both groups of participants, with and without training (G1 and G2, respectively). The t-test analysis shows a significant training effect on the surface area of the CoP. Indeed, for G1 only, the surface area of the CoP decreases significantly after training in the perturbed vision condition with optocinetic stimulation on unstable support (p<0.03). The t-test analysis shows that the surface area of the CoP for G2 during the second postural measure increases significantly (p<0.01).

#### Mean velocity of the CoP

**Fig 2:**
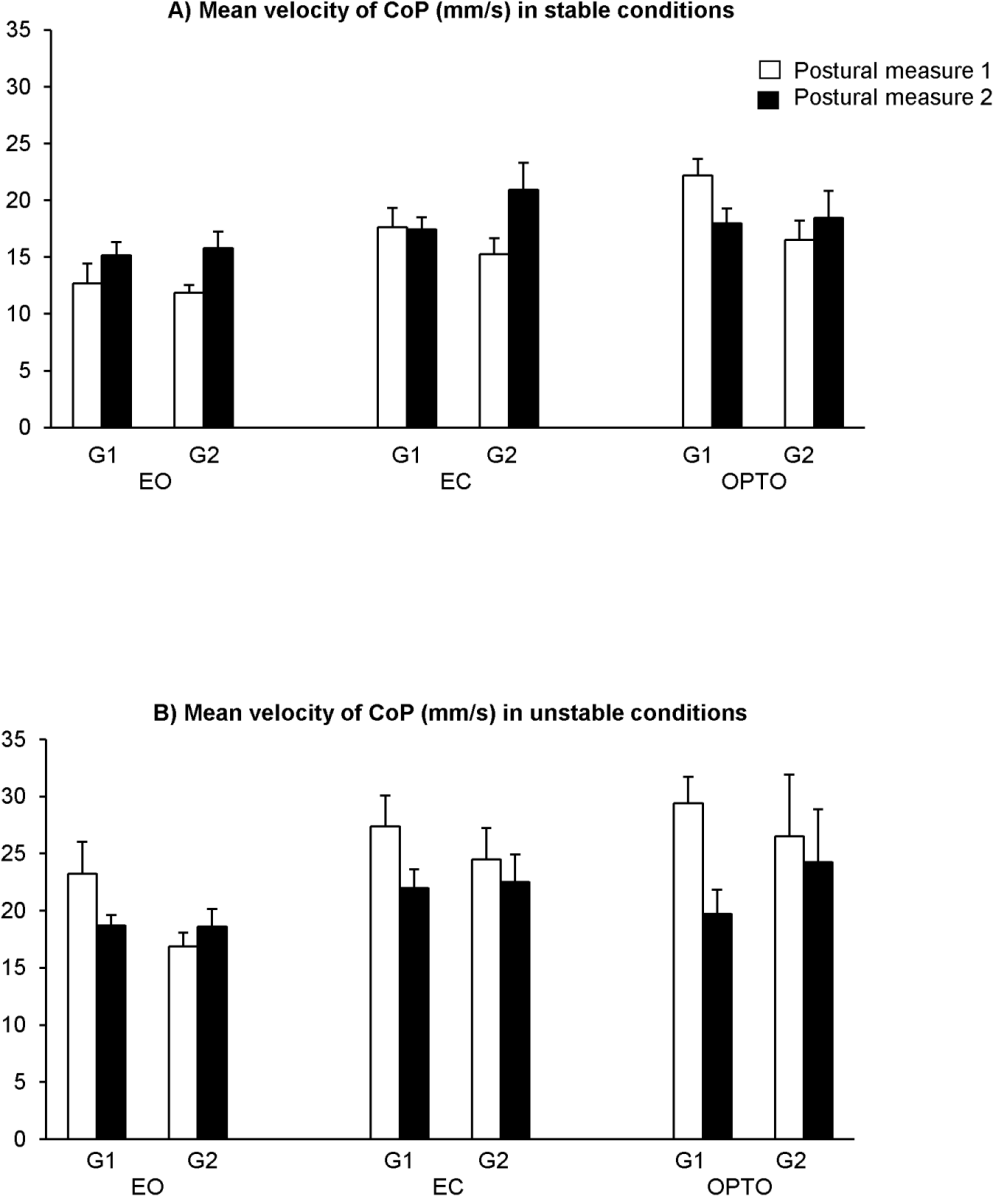
Means and standard deviations of mean velocity of CoP (mm/s) in all conditions tested (EO, EC and OPTO in stable condition (A) and in unstable condition (B) during the two postural measures (1 and 2) for both groups of children with (G1) and without training (G2).

Fig 2 shows the mean velocity of the CoP (mm/s) in all conditions tested (EO, EC and OPTO) in stable condition (Fig 2A) and in unstable condition (Fig 2B) during the two postural measures (1 and 2) for both groups of participants with (G1) and without training (G2). For G1, the t-test analysis shows a significant training effect on the mean velocity of the CoP: its value decreases significantly after training in G1 in the perturbed vision condition with optocinetic stimulation on unstable support surface (p<0.01). For G2, the t-test does not show any statistical difference between the two postural measures.

### Temporal analysis, wavelet transformation

#### Spectral power indices in medio-lateral and antero-posterior direction

**Fig 3:**
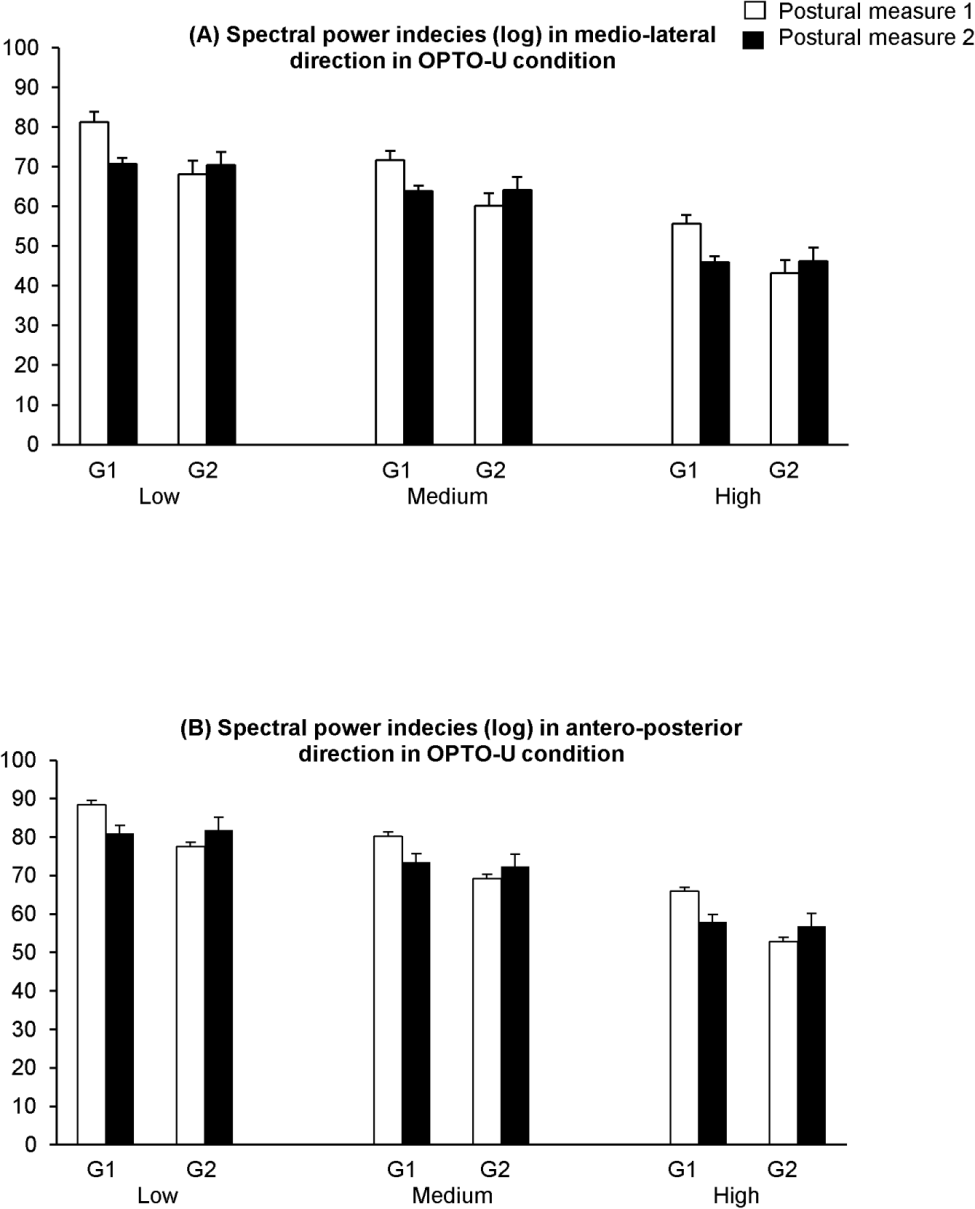
Means and standard deviations of spectral power indices (log) in medio-lateral (PIx) (A) and antero-posterior direction (PIy) (B) in perturbed vision with optocinetic stimulation in unstable condition (OPTO-I) in all frequencies: low (L), medium (M) and high (H), during the two postural measures (1 and 2) for both groups of children with (G1) and without training (G2).

Fig 3 shows the spectral power indices (log) in medio-lateral (PIx) (Fig 3A) and antero-posterior directions (PIy) (Fig 3B) in perturbed vision with optocinetic stimulation in unstable condition (OPTO-U) in all frequencies: low (L), medium (M) and high (H), during the two postural measures (1 and 2) for both groups of participants, with training (G1) and without training (G2).

The t-test analysis shows for G1 a significant training effect on spectral power indices in the medio-lateral direction: its value decreases significantly after training for all frequencies (low, medium and high frequency) in perturbed vision with optocinetic stimulation on unstable support surface (p<0.01, p<0.02 and p< 0.01, respectively). In contrast, for G2, the t-test analysis shows a significant increase of spectral power indices in medio-lateral direction with eyes open on stable support surface in medium and high frequencies (both p<0.03) and with eyes closed in all frequencies (p<0.02, p<0.01, p<0.01, respectively). Also, the t-test analysis shows a significant increase of spectral power index with eyes open on unstable support surface for high frequency (p<0.03).

Moreover, the t-test analysis shows for G1 a significant training effect on spectral power indices also in the antero-posterior direction. Indeed, its value decreases significantly in G1 after training in perturbed vision condition with optocinetic stimulation on unstable support surface for all frequencies (p<0.04, p<0.04 and p<0.02, respectively). For G2 the t-test does not show any statistical difference in the antero-posterior direction between the two postural measures.

#### Cancelling time in medio-lateral and antero-posterior direction

**Fig 4:**
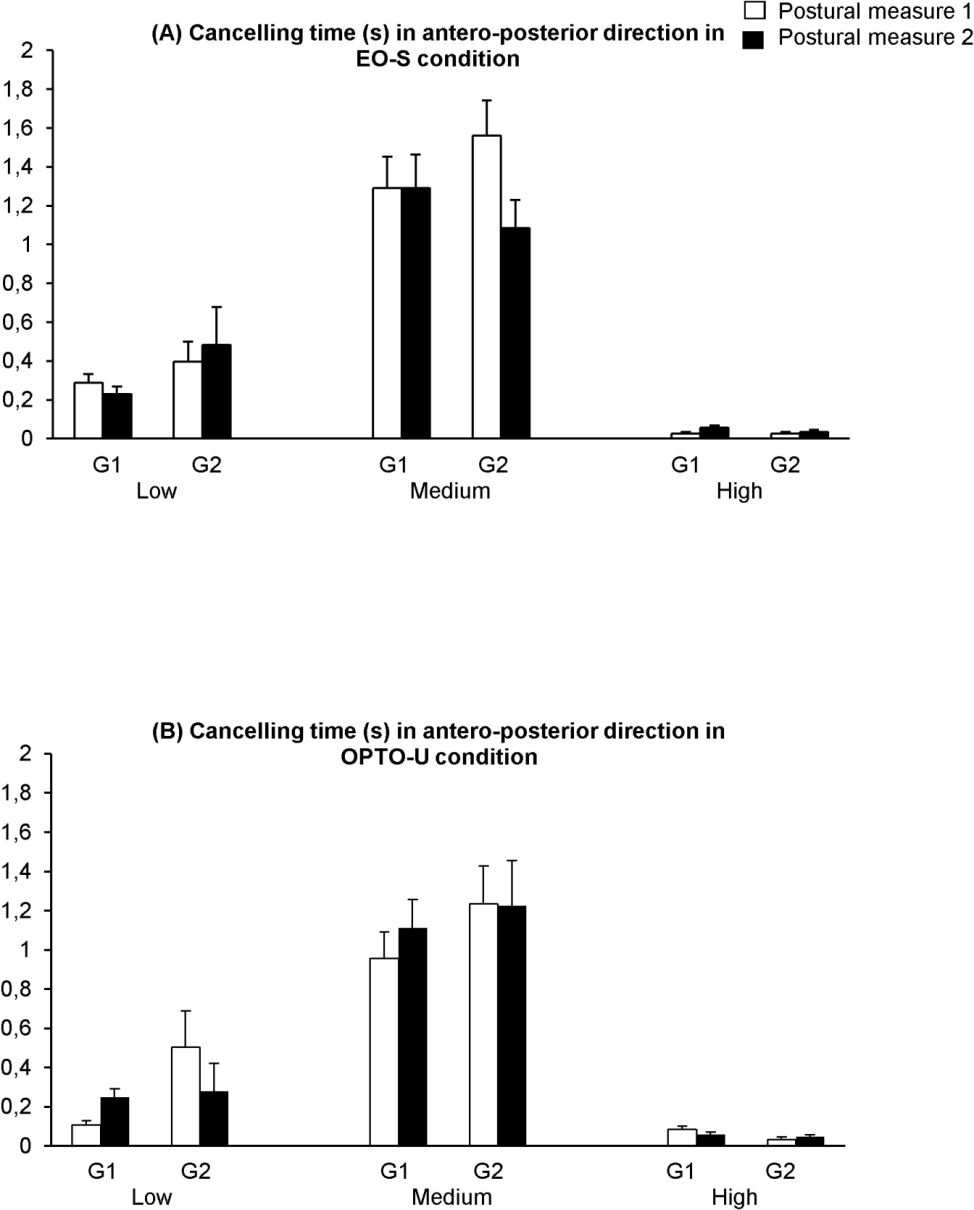
Means and standard deviations of the cancelling time (s) in antero-posterior direction (CTy) with eyes open in stable condition (EO-S) (A) and in perturbed vision with optocinetic stimulation in unstable condition (OPTO-U) (B) in all frequencies: low (L), medium (M) and high (H), during the two postural measures (1 and 2) for both groups of children with (G1) and without training (G2).

Fig 4 shows the cancelling time (s) in antero-posterior direction (CTy) with eyes open in stable condition (EO-S) (Fig 4A) and in perturbed vision with optocinetic stimulation in unstable condition (OPTO-U) (Fig 4B) in all frequencies: low (L), medium (M) and high (H), during the two postural measures (1 and 2) for both groups of participants, with (G1) and without training (G2).

The t-test analysis on the cancelling time in medio-lateral direction does not show any statistical difference for G1; in contrast, for G2 the t-test analysis shows a significant decrease with eyes closed on stable support surface for low frequency only (p<0.03).

The t-test analysis on the cancelling time in the antero-posterior direction shows for G1 a significant training effect on; its value increases significantly in the eyes open condition on stable support for high frequency only (p<0.02). Also, the cancelling time increases significantly in the perturbed vision with optocinetic stimulation on unstable support surface for low frequency only (p< 0.01). In contrast, for G2, the t-test analysis shows a significant decrease of cancelling time with eyes open on stable support surface for the medium frequency only (p<0.01).

## Discussion

The main findings of this study are as follows: (i) Short postural training decreased the surface and the mean velocity of CoP when vision is perturbed with optocinetic stimulation in the unstable condition; (ii) After short postural training the parameters of temporal analysis of the CoP changed, suggesting an improvement in postural control. Each of these findings will be discussed below.

Firstly it is important to note that for the second group (G2) of dyslexic participants (without postural training) few postural variables significantly changed but not in the corrected way. Indeed, in the second postural recording G2 participants showed a significant increase of the surface area with eyes closed on stable support surface, a significant increase of the spectral power indices and a decrease of the cancelling time. Such changes are in line with Dumistrescu and Lacour [32] and Bernard Demanze et al. [27]. Indeed, our result showed that postural control measured the second time was worse than postural control measured the first time. Such changes for G2 are most likely due to fatigue and to poor capabilities to maintain balance in dyslexic participants. Consequently we can believe that the improvement found after the short postural training for G1 could be due to the effect of the short postural training. Recall that both G1 and G2 had a rest time between the two postural recordings in order to avoid possible fatigue effect.

(i) Short postural training decreased the surface and the mean velocity of the CoP when vision is perturbed with optocinetic stimulation in the unstable condition.

Our results showed that postural control during perturbed vision with optocinetic stimulation in the unstable condition improved significantly after a short postural training. According to Woollacott et al. [38], training could improve motor control efficiently, leading to better use of muscular activities in dyslexic children and thus to improved postural control [30–31]. Maintaining postural control with a low cost of muscular activity is important on a daily basis to achieve efficiently postural stability with lower energy cost in order to reduce fatigue effects. Postural training could allow a better control of the functional degree of freedom between muscles and joints, leading to a better motor coordination involved in postural stability. The work by Faigenbaum et al. [39] compared in a large sample of 188 children (from 6.9 to 12.1 years old) the effect of an 8-week-long muscular training. Their results showed that muscular training is characterized by an improvement of performance during several exercises (abdominal curl-up and single leg hop). Another study by Zech et al. [40] on 30 adolescent athletes (mean age 14.9 years) explored the effect of 20 minutes twice a week of muscular training. Interestingly, after ten weeks of muscular training participants showed greater improvement in postural capabilities in comparison to controls who were not trained.

Finally one has to point out that improvement occurs when the participant is in the most difficult condition (unstable platform with pertubed vision). Maybe training done several times per week could improve postural stability also in the easier conditions. Another study testing this hypothesis on a large population of dyslexic participants should be conducted.

(ii) After a short postural training, the parameters of temporal analysis of the CoP changed, suggesting an improvement in the postural control.

The results on wavelet transformation showed an improvement in the postural control of dyslexic participants after training. Such improvement could be due to training, capable to stimulate participants to use all the sensory information which they receive and to better integrate them via the cerebellum. The improvement with training in dyslexic participants could take place via brain plasticity. Indeed, several studies showed that a short training period is capable to produce structural brain reorganization with namely an increase of grey matter in parietal and frontal cortical areas in adult subjects (25 years) [41]. These authors showed that in 28 young adults (25 years old), after six weeks of this training, the grey matter increased in the pre-frontal area, thus suggesting the plasticity of the human cortex. Importantly, these authors observed that the most prominent performance changes occurred at the beginning of the learning phase, consequently even a short training period could have an impact on brain plasticity and thus on the learning process. A recent study by Burciu et al. [20] using EEG, in a large sample of adult subjects from 26 to 73 years, provided the additional evidence that a short period, two weeks, of sensory-motor training enhanced motor performance in patients with cerebellar degeneration. These authors identified training-related changes with an increase of gray matter in the pre-motor cortex and in the cerebellum (both involved in postural control). Thus, this change allowed by cortical plasticity could be involved independently to the age of the subject. Furthermore, as already mentioned in the introduction, Sehm et al. [19], using MRI, showed that in Parkinson’s disease fast structural changes were found already at the beginning of training, with an increase of grey matter in the left inferior parietal cortex then in the left cerebellum and in the right inferior temporal gyrus. Although this study has been conducted in older people with Parkinson’s disease, we need to recall that a cerebellar deficit is also reported in dyslexic children [3–9; 15–21]; consequently cerebellar plasticity could hypothetically also occur in dyslexic participants, leading to improved postural control.

Finally, we have to mention that several studies [42–44] showed that postural improvement of motor function is maintained until one year after training after a longer period of training (8 weeks). Thus a shorter training period as used in this study could have a shorter effect in maintaining good postural abilities. Further studies examining the persistence of the effect of training in a longitudinal study are needed, along with a test of reading capabilities before and after training. Indeed, no correlation between postural measures and reading abilities in dyslexic participants has been found. Note, however, that a work by Reynolds et al. [45] explored the effect of eye movements, motor and postural exercices in 35 young children (3 years old) with risk of reading difficulty. They showed that the children who performed the training programme twice a day improved not only their postural abilities but also their cognitive skills (as dexterity, reading, writing process, verbal and semantic fluency). This study suggests that after training, a general improvement occured in central structures controlling motor as well as cognitive abilities.

## Conclusion

Our current study showed that a short postural training period could improve postural control in dyslexic participants. Such training enhances postural capability due to a relevant use of a muscular pattern to achieve efficiently the postural control, maybe via brain plasticity, which allows better performance in both sensory process and cerebellar integration. Indeed, training could allow a better sensory input processing due to an increase of the neural network, leading to improved postural stability. Further studies on a large sample of dyslexic participants with different types of training could improve our knowledge on how training affects postural control in this kind of children population. Moreover, such type of postural training could be developed in other disorders with cerebellar etiology.

## Supporting Information

S1 Data (XLSX)

S1 Movie Movie showing the short postural training period used by the group G1 of dyslexic children. (ZIP)
